# Rest‐to‐work and work‐to‐rest transients of interstitial PO_2_ in spinotrapezius muscle of young and old male rats

**DOI:** 10.14814/phy2.70260

**Published:** 2025-02-27

**Authors:** Aleksander S. Golub, William H. Nugent, Roland N. Pittman, Bjorn K. Song

**Affiliations:** ^1^ Song Biotechnologies LLC Baltimore Maryland USA; ^2^ Department of Physiology and Biophysics Medical College of Virginia at Virginia Commonwealth University Richmond Virginia USA

**Keywords:** aging, exercise, microcirculation, oxygen transport, skeletal muscle

## Abstract

Muscle function declines with age. Since the primary energy source for contraction is aerobic, this study investigated age‐related changes in muscle oxygenation dynamics to: characterize PO_2_ transients during rest‐work transitions, identify age‐specific differences in oxygen delivery/utilization balance, and examine the relationship between interstitial and arterial oxygen tension (PO_2_). Interstitial PO_2_ was measured with a high‐resolution stroboscopic phosphorescence quenching technique to map intra‐contractile dynamics during changes in muscle activity—rest‐to‐work (RtW) and work‐to‐rest (WtR) in rats aged three (young) and 23 (old) months. RtW (τ_w_) and WtR (τ_r_) PO_2_ transitions had lag periods and mono‐exponential time constants. In young muscles, lag was 4 s, τ_w_ = 9.0 ± 3.7 s, and τ_r_ = 15.4 ± 3.9 s. For old, lag was also 4 s with increases to τ_w_ = 15.9 ± 3.5 s and τ_r_ = 41.4 ± 8.3 s. Resting PO_2_'s were higher for young than for old (66.7 ± 13.7 vs. 60.2 ± 13.0 mmHg; *p* < 0.05). Work reduced PO_2_ with a greater effect on old (42.5 ± 14.0 vs. 28.3 ± 16.5 mmHg; *p* < 0.05). Intra‐contractile measurements revealed a spike in PO_2_ (11 mmHg amplitude for >200 ms), which was absent in old. Further, sustained exercise in young showed a rising trend in PO_2_, while old remained at nadir. The missing PO_2_ spike in aged muscle contributes to reduced PO_2_ during work and may explain age‐related loss of endurance.

## 1 INTRODUCTION

Aging is characterized by a progressive decline in physiological function, with skeletal muscle performance a vital indicator of this process. The deterioration of strength and endurance significantly impacts mobility and independence, leading to complex psychological, social, and medical issues, particularly in humans. While sarcopenia, the age‐related loss of muscle mass and strength, is associated with reduced neuromuscular contact density and changes in muscle fiber composition (Dao et al., 2020; Demontis et al., 2013; Larsson et al., 2019), there is a concomitant reduction in metabolism and exercise capacity that is a subject of intensive research.

Two primary hypotheses address the reduced bioenergetics of senescent skeletal muscle: (1) Vascular remodeling and rarefaction decrease oxygen (O_2_) delivery to myocytes (Bearden, 2006; Muller‐Delp, 2016; Payne & Bearden, 2006; Ungvari et al., 2010, 2018); and (2) Reduced O_2_ consumption by senescent mitochondria (Amorim et al., 2022; Austad, 2018; Demontis et al., 2013). These theories are not mutually exclusive, as they focus on two separate but serially linked components of the O_2_ supply/demand chain. Further, a component of declining function may be due to coordination between microvascular remodeling and mitochondrial volume attempting to maintain a balance between O_2_ delivery and consumption. This potential aging mechanism combines both primary hypotheses and can be viewed as an “adaptive” aging hypothesis. However, in old muscles, the adaptive balance between O_2_ supply and consumption in a resting state may not have sufficient capacity to persist during muscular work.

Skeletal muscle demonstrates remarkable plasticity in response to both use patterns and oxygen (mainly arterial PO_2_) availability. This adaptive capacity involves coordinated changes in vascular supply and metabolic demand that become less flexible over the lifespan due to the interplay of senescing myofibers and their mitochondria and the microvascular network in parallel. “Adaptive” aging seeks to maintain the balance between O_2_ supply and O_2_ utilization at rest and under working conditions. Therefore, the interstitial PO_2_s at rest and during work, respectively, should not change with age despite microvascular remodeling and reduced mitochondrial capacity. If interstitial PO_2_ does change, it reflects an imbalance between the two systems and may provide insight into aging's leading edge. For example, arterial blood PO_2_ decreases with age (Blom et al., 1988; Delclaux et al., 1994; García‐Río et al., 2007) due to a pulmonary mechanism, which suggests a supply origin for declining muscle function. If resting interstitial PO_2_ declines as a result, then a maladaptive imbalance exists within the aging muscle.

There is agreement that capillary diameter does not change with muscle aging (Golub & Brod, 1993; Kano et al., 2002; Russell et al., 2003). It has also been noted that the density of perfused capillaries decreases with age (Golub & Brod, 1993; Russell et al., 2003; Sarelius et al., 1981). However, according to some data, the blood velocity in capillaries is reduced in old muscles (Golub & Brod, 1993; Sarelius et al., 1981); according to other data, it remains unchanged or even increases (Copp et al., 2009; Russell et al., 2003). Capillary hematocrit drops with age (Sarelius et al., 1981) or remains unchanged (Copp et al., 2009; Golub & Brod, 1993; Russell et al., 2003). Regarding the decline in mitochondrial oxidative capacity, there is a consensus in the literature supported by experimental data (Hagen et al., 2004; Hepple et al., 2003). However, experimental evidence that does not support the mitochondrial theory of aging is also available (Conley et al., 2000; Rasmussen et al., 2003).

Additional study is needed on the dynamics of interstitial PO_2_ in skeletal muscles of different ages at rest and under workload. The development of the phosphorescent quenching method (Vanderkooi et al., 1987) made noninvasive measurement of the PO_2_ in various compartments of active organs possible. Early studies investigated the spinotrapezius muscles from young and old rats through intravascular PO_2_ that was measured using phosphorescent signals from arterial and venous microvessels (Russell et al., 2003). These early measurements of microvascular PO_2_ (33.8 vs. 29.1 mmHg, young vs. old, respectively) paved the way for long‐term studies of spinotrapezius muscle at this level of PO_2_ (Behnke et al., 2002, 2006, 2007; McCullough et al., 2011). In the same experiments, O_2_ consumption in the spinotrapezius muscle was measured, and it was found to be negligible between young and old muscles.

The present study is part of our systematic investigation of muscle oxygen delivery and utilization dynamics. Our previous work established methods for measuring oxygen consumption of the spinotrapezius muscle in situ (Golub & Pittman, 2012; Smith et al., 2002), characterized the oxygen dependence of muscle respiration (Golub et al., 2018), and examined interstitial PO_2_ dynamics and steady states during rest and exercise (Golub et al., 2023). These studies led to the development of an electrical analog model (Golub et al., 2018) that describes the mechanism controlling the balance between oxygen delivery and utilization. This approach allows for the comparative analysis of metabolic and transport functions in young and old muscles, utilizing data on the dynamics of interstitial PO_2_ during rest and exercise. By comparing age groups, we aim to identify how the oxygen delivery–utilization balance changes with aging, providing both direct observations of oxygen dynamics and experimental validation for theoretical frameworks.

Subsequently, the study of O_2_ balance in the spinotrapezius muscle was enhanced by studying the “kinetics” of microvascular PO_2_ when a workload test was applied to the muscle (Behnke et al., 2006). Important experimental variables were then identified as PO_2_ at rest and at “nadir” under load, time constants for onset and offset transients, time lag, and undershoot (Behnke et al., 2002, 2006, 2007; McCullough et al., 2011).

Furthering these approaches, we used a stroboscopic measurement mode for phosphorescence quenching microscopy and reported the PO_2_ transients evoked by a spinotrapezius muscle contraction with a 20 ms temporal resolution (Golub et al., 2021). Their physiological significance for O_2_ transport in contracting muscles is further explored in this report, but also reflects some intimate mechanisms of vascular‐to‐muscular interactions.

The work presented here aimed to obtain characteristics of PO_2_ balance in normoxic resting and working muscles for young and old normal rats, using the interstitial PO_2_ compartment to indicate the balance of O_2_ delivery and utilization. Another aim of this work was to compare interstitial PO_2_ transients of muscles from young and senescent rats using standard muscle contraction on/off protocols in combination with a novel stroboscopic approach. In addition, we tested the hypothesis that the O_2_ supply/O_2_ utilization system was adaptive to aging in the sense that the simple calculation of the interstitial PO_2_/arterial PO_2_ ratio at rest and work remained relatively constant, taking into account the different arterial blood PO_2_ in young and old rats.

## 2 MATERIALS AND METHODS

### 2.1 Animal model and ethical statement

SoBran Biosciences Inc. (Baltimore, MD) approved the following animal IACUC protocol, ethic, and experimental procedures (Protocol #SON‐005‐2018) executed by Song Biotechnologies LLC (Baltimore, MD) researchers. All procedures involving animals were consistent with ethical standards set by the National Institutes of Health Guidelines for the Humane Treatment of Laboratory Animals and the American Physiological Society's Guiding Principles in the Care and Use of Animals. Six young adult rats (young, 3 months old) and six aged rats (old, 23 months old) male Sprague–Dawley rats (Harlan, Indianapolis, IN) were used in the muscle contraction experiments (Table 1).

**TABLE 1:** Physiological characteristics of young and old rats.

Animal parameters	Young (*N* = 6)	Old (*N* = 6)
Age (months)	3, emerging adulthood	23, older adulthood
Body weight (g)	305 ± 25	832 ± 227*
Mean arterial pressure (mmHg)	117 ± 10	96 ± 24 (*p* = 0.08)
Heart rate (bpm)	433 ± 24	362 ± 44*

*Note*: Data are presented as mean ± SD. Young: 3 months old (*N* = 6); Old: 23 months old (*N* = 6).

* *p* < 0.01 between mean values for age groups.

### 2.2 Surgical preparation

Animals were induced with 1%–5% isoflurane delivered in medical air for initial preoperative preparation and cannulations. The femoral vein was then accessed and cannulated with polyethylene tubing (PE‐90) to enable the continuous infusion of anesthetic, alfaxalone acetate (Alfaxan; Schering‐Plough Animal Health, Welwyn Garden City, UK), at a rate of ~0.1 mg/kg/min. The infusion rate was adjusted based on animal reflexes, heart rate, and O_2_ saturation indicators and provided a steady plane of anesthesia through the conclusion of surgical preparation and measurement. A tracheal tube was inserted to maintain a patent airway, and animals were allowed to spontaneously breathe room air. A femoral artery was cannulated to monitor hemodynamics with a multichannel physiological monitoring system (BIOPAC MP‐150; BIOPAC Systems, Goleta, CA). Body temperature was maintained at 37 ± 0.2°C by a custom 3D‐printed microscopic platform (Golub & Pittman, 2003) and a rectal temperature probe. The main parameters characterizing the physiological status of the two groups of animals are listed in Table 1. After completing experimental measurements, animals were euthanized with Euthasol (360 mg/mL pentobarbital and 50 mg/mL phenytoin sodium administered I.V. at 3 mL/kg; VetOne; Boise, ID).

### 2.3 Spinotrapezius muscle preparation

The rat spinotrapezius muscle was prepared as previously described (Gray, 1973), with modifications for isometric contraction measurements. The exteriorized muscle was placed on a transparent, thermostatically controlled pedestal at 36.5°C. Seven sutures secured the muscle edges to a rigid frame to minimize movement during contractions. Two chlorinated silver wire electrodes were attached along the muscle's side edges for electrical stimulation. A brief (1–5 s) stimulation was applied to ensure proper muscle fixation and electrode connection.

The muscle stabilized for 20 min while the O_2_‐sensitive probe—liquid—was loaded into the interstitium (Golub et al., 2011). The probe, Pd(II) meso‐Tetra(4‐carboxyphenyl)porphine (PdT790; Frontier Scientific, Newark, DE), was conjugated to bovine serum albumin as previously described (Smith et al., 2002) to limit counter‐flow diffusion into the vasculature. The muscle was covered with the gas barrier film (Krehalon, CB‐100, Kureha Corporation, Tokyo, Japan) to prevent tissue desiccation and interference from atmospheric O_2_.

An objective‐mounted airbag made of transparent film (Krehalon) was inflated to provide 8 mmHg organ compression, ensuring free blood circulation while preventing fluid accumulation between the film and tissue (Golub et al., 2011).

### 2.4 Phosphorescence quenching microscopy

The choice of interstitial PO_2_ measurements, rather than alternative approaches, was deliberate. Interstitial PO_2_ represents the critical interface between oxygen delivery and utilization, integrating both vascular supply and metabolic demand.

Interstitial PO_2_ measurements (Nugent et al., 2016) were performed using a fluorescence Axioimager‐A2m microscope with a 20X/0.8 Plan‐Apochromat objective lens (Carl Zeiss, Germany). The excitation light source was a 520 nm green laser diode (NDG7475 1W; Nichia Tokushima, Japan) powered by a pulse laser driver iC‐HKB (www.ichaus.com). An optical cube in the path of the epi‐illumination train contained a dichroic beam splitter (567 nm DMLP) and an interference filter (Cut‐on >650 nm, Thorlabs, Newton, NJ) for emitted phosphorescence. The circular excitation region (450 μm diameter) covered more than 10 fibers (width of fiber = 33.6 ± 3.3 μm; 22 areas measured). The laser pulse duration was 1 μs, delivering 8 pJ/μm^2^ to the muscle surface. The combination of a low probe concentration and low excitation intensity resulted in acceptable O_2_ consumption by the method (0.4% of PO_2_ per excitation flash) (Golub et al., 2018). PO_2_ was calculated using exponential analysis of phosphorescence decay curves and a fitting model for heterogeneous decay curves (Golub et al., 1997; Golub & Pittman, 2016). Data was acquired and processed using a custom‐built Virtual Instrument in LabView (National Instruments: NI.com).

### 2.5 Muscle stimulation and measurement protocol

Studies of O_2_ kinetics at rest and work are best performed in the interstitial compartment of skeletal muscle (Golub et al., 2018, 2023; Hirai et al., 2018, 2019; Nugent et al., 2016) because it is a focal point of gas exchange between the vasculature and the tissue.

The literature uses two sets of terms to denote the transients under study: (1) Rest‐to‐Work (RtW) and Work‐to‐Rest (WtR) transitions (Wilson, 2015), and (2) On‐set and off‐set of exercise transitions (Behnke et al., 2002; Paterson & Whipp, 1991). We have utilized the first convention to describe the transitions in our study and the term “nadir” (Hirai et al., 2019) to denote the lowest PO_2_ point during the RtW transition.

The built‐in electrical stimulator produced symmetrical 10‐volt and 20‐ms pulses delivered to a pair of muscle electrodes. Measurement sites were clustered in the central part of the muscle to minimize contractile displacement. Excitation laser pulses were targeted to muscle regions between transverse arterioles. This ensured that interstitial PO_2_ was measured in the space between capillaries and muscle cells.

A rectangular wave workload test was carried out by recording onset and offset transients in different regions of the same muscle. We used the obtained transients to characterize the O_2_ delivery and consumption in the muscles of young and old rats. The goal was to determine how aging affects the O_2_ kinetics of RtW, WtR, and within a muscle contraction. Experimental data on the values and rates of the PO_2_ transitions between rest and work are necessary to develop a mathematical model of the interaction between systems providing O_2_ delivery and utilization in the muscle.

**FIGURE 1:**
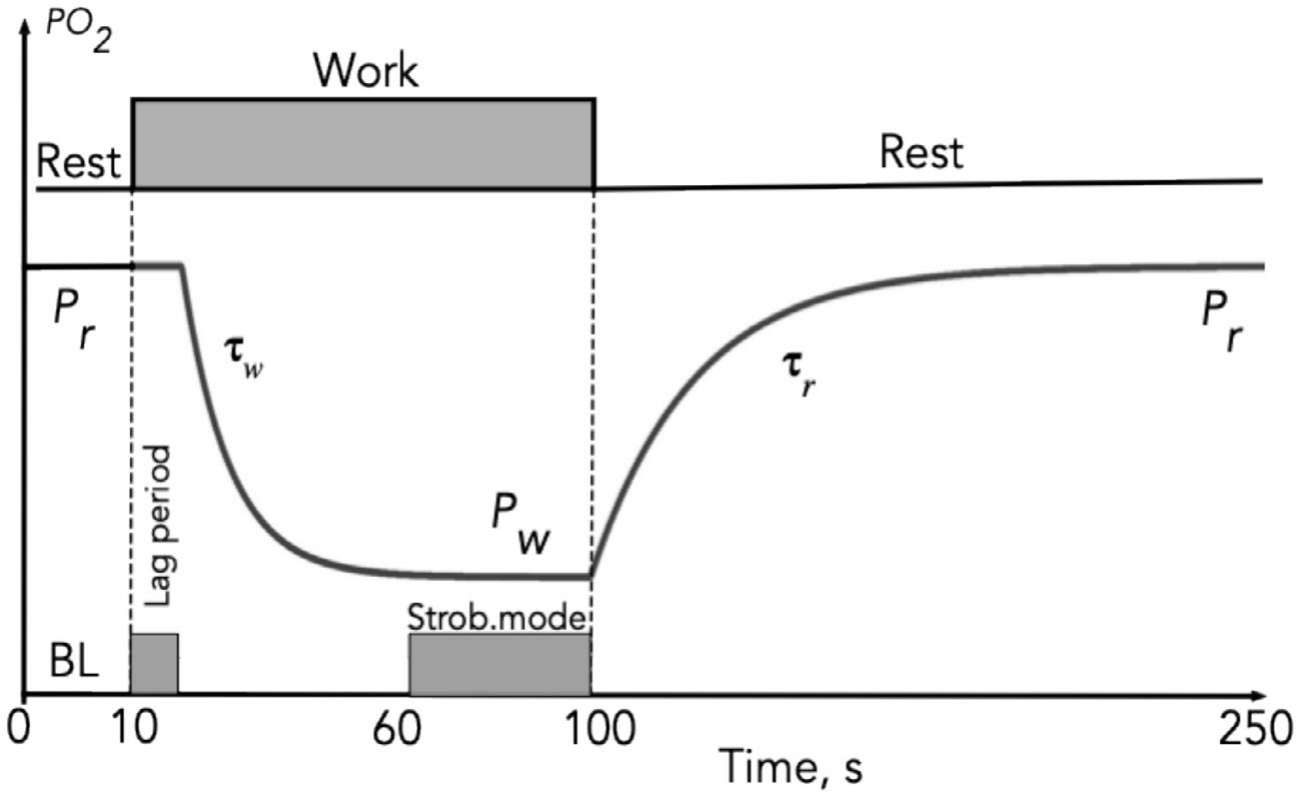
Experimental design and conceptual PO_2_ response to muscle contraction test. A conceptual diagram of the time course of the interstitial PO_2_ in response to a rectangular wave of workload. Explanation of the measured parameters: *P*_r_ is baseline PO_2_ and asymptotic PO_2_ at resting steady state; *P*_w_ is asymptotic PO_2_ for work transients or lowest PO_2_ at work (nadir). The transition of the rest‐to‐work model shows a lag period (time delay) before the exponential decline in PO_2_ begins. For experimental curves, this lag is not flat. Next, PO_2_ decreases at a rate determined by the time constant for the work (τ_w_). Recovery of resting level PO_2_ is characterized by a time constant for resting muscle (τ_r_).

The experimental time course was divided into 250 one‐second intervals, and the order of PO_2_ sampling was controlled by an Arduino Uno R3 microcontroller (https://www.arduino.cc/). Each measurement series was preceded by at least 4 min of rest. The protocol included (see Figure 1):

1. Baseline PO_2_ measurements (10 s)
2. Rest‐to‐work transition (50 s)
3. Stroboscopic mode measurements (40 s)
4. Work‐to‐rest transition and recovery (150 s)

The first 10 PO_2_ values (#1–10 s) were recorded without electrical stimulation as a baseline (BL) PO_2_ or *P*_r_. From flash #11 to #60, the electrical stimulation pulses were followed by a laser flash, so the PO_2_ was sampled before the muscle twitch.

During the stroboscopic mode (from sample #61 until #100), the phase of the laser flash was progressively delayed by 20 ms increments from the front of the stimulator pulse, allowing high‐resolution recording of PO_2_ dynamics during 800 ms of a serial muscle contraction (Golub et al., 2021).

Starting from sample #101 and up to #250, the muscle was in a state of rest and recovery of the interstitial PO_2_. After a 1‐min interval, another 250‐s protocol began on the same (up to five replicates) muscle site or a new one. A comparison of average PО_2_'s (*P*_r_) at BL #1–10 and the end of recovery #241–250 was used to characterize the completeness of PO_2_ recovery.

### 2.6 Statistics

In each experiment, 4–5 PO_2_ recordings were measured per site with a range of 4–5 different sites per spinotrapezius muscle. Collectively, 117 young muscle sites and 139 old muscle sites were part of this study. Descriptive statistics and paired *t*‐tests were used from the Data Analysis Tools in Microsoft Excel (Microsoft Corporation, Redmond, WA). All data in the text and tables are presented as mean ± SD, while data in the figures are presented as mean ± CI 95%. Statistical significance for the difference between mean values was set at *p* < 0.05.

## 3 RESULTS

### 3.1 Animal characteristics and baseline physiological parameters

Two significant differences existed between young and old: Older rats were heavier and their heart rates were lower (Table 1). Mean arterial blood pressure was also reduced by an average of 21 mmHg but fell short of significance at (*p* = 0.08). Old rats also exhibited a more heterogeneous body weight and presented with a senile appearance and increased adiposity.

### 3.2 Interstitial PO_2_ dynamics

**FIGURE 2:**
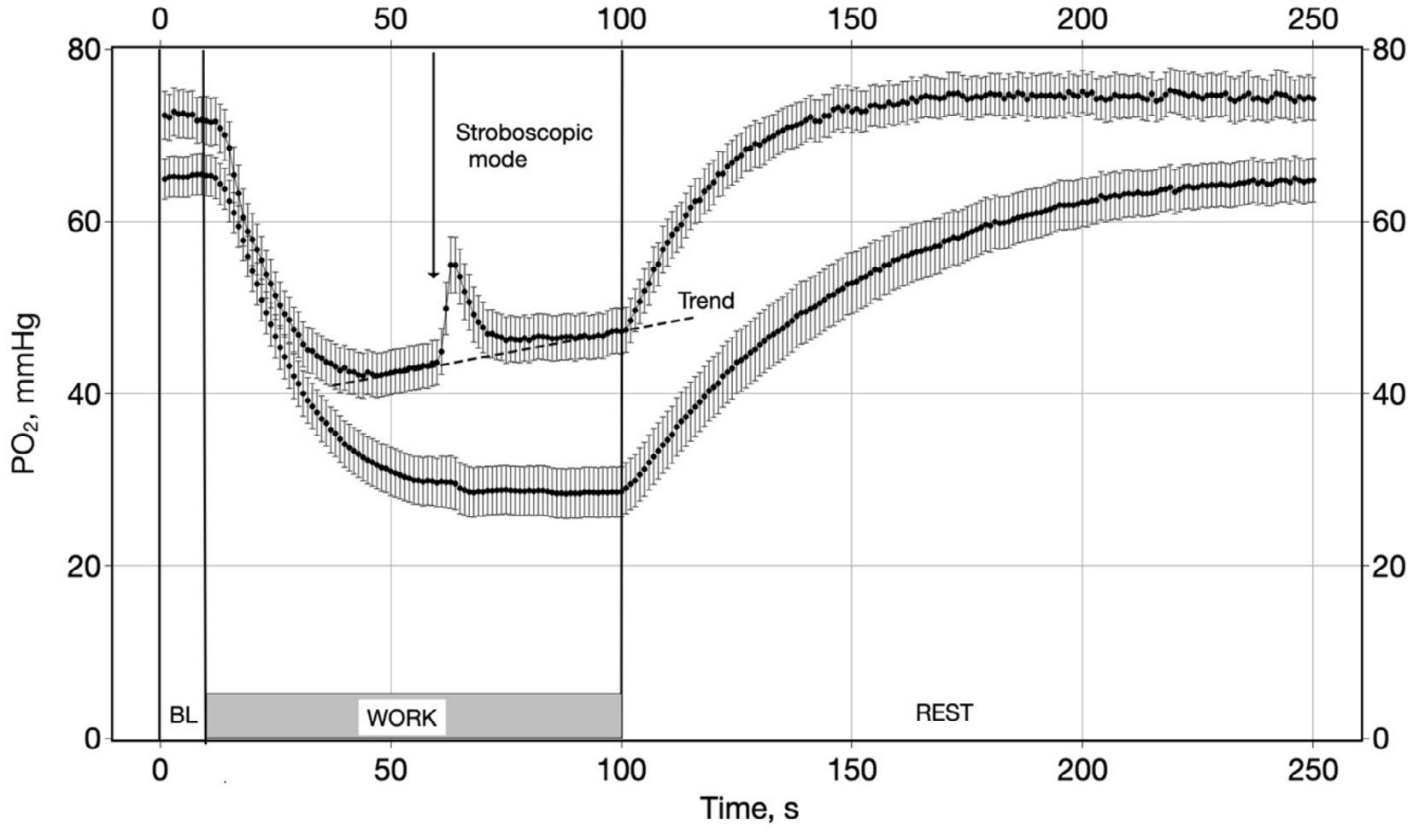
Interstitial PO_2_ dynamics during rest‐to‐work and work‐to‐rest transitions in young and old rat muscles. PO_2_ response during RtW, contraction, and WtR. These are experimental results on interstitial PO_2_ in young (top line) and old (bottom line) rats during work and rest periods. Data are presented as mean ± CI 95%. Baseline (BL) PO_2_ was average for measurements #1–10 s. Stimulated muscle contractions (work period) lasted from #11–100 s; muscle rest and PO_2_ recovery period started at #101 and lasted to #250 s. In the time interval from #61–100, PO_2_ measurements were made with a progressive lag with respect to the electrical stimulation pulse. This stroboscopic method reveals the PO_2_ time course during stationary work with a time resolution of 20 ms. The dashed line in a young muscle is a positive PO_2_ trend, approximated as 37.7+ 0.096 *t (s).

Interstitial PO_2_ measurements during rest‐to‐work (RtW) and work‐to‐rest (WtR) transitions revealed distinct patterns in young and old muscles (Figure 2). Both age groups exhibited a brief delay (4 s) in the PO_2_ response at the onset of muscle contractions (RtW). However, no delay was observed in the rising PO_2_ response during WtR. The experimental curve in young was characterized by an intra‐contractile PO_2_ spike, as previously described (Golub et al., 2021), and a positive PO_2_ trend, which were, strikingly, absent in old.

To determine the resting O_2_ levels, we averaged 10 data points before the onset of stimulation and 10 points at the end of recovery. The resulting averages were used for further statistical analysis as one value (*P*_r_) characterizing PO_2_ in a site (Table 2). The comparison of recovered O_2_ pressure with baseline PO_2_ indicated that a 150‐s rest period was enough for the muscles to recover their oxygenation after 90 s of exercise (Figure 2). The average O_2_ levels at rest, during exercise, and at the end of the recovery period are in Table 2.

**TABLE 2:** Interstitial PO_2_ values at rest and during muscle contraction in young and old rats.

Measurement point	Young (*N* = 117)	Old (*N* = 139)
	mmHg	mmHg
*P*_r_ (rest PO_2_, #1–10)	66.7 ± 13.7	60.2 ± 13.0*
*P*_r_ (recovered PO_2_, #241–250)	68.7 ± 13.0	59.8 ± 14.0*
*P*_w_ (work PO_2_, nadir)	42.5 ± 14.0	28.3 ± 16.5*

*Note*: Data are presented as mean ± SD. *N* represents the number of measurements in muscle sites. Ten PO_2_ measurements at rest on each experimental curve were averaged for 117 (Young) and 139 (old) recorded curves. During the period of work, only one lowest PO_2_ point *P*_w_ (nadir) was taken for each recorded curve for averaging.

Abbreviations: *P*_r_, resting PO_2_; *P*_w_, PO_2_ during muscle contraction (nadir).

* *p* < 0.001 between mean values.

### 3.3 Rest‐to‐work and work‐to‐rest transitions

Baseline PO_2_ (*P*_r_) in old was 6.5 mmHg lower (*p* < 0.001) than young. PO_2_ in older muscles was also lower after recovery by 8.9 mmHg (*p* < 0.001). The differential expanded during muscle contraction at the PO_2_ nadir point by 14.2 mmHg (*p* < 0.001; Table 2).

To estimate the rate of transitions between states of rest and work, we used a mono‐exponential approximation characterized by a time constant. The results of fitting PO_2_ transients in Figure 2 are shown in Table 3. The time constants for RtW (τ_w_) and WtR (τ_r_) transitions differed significantly between age groups. The PO_2_ in young muscles exhibited faster RtW transitions (τ_w_ = 9.0 s) than in old muscles (τ_w_ = 15.9 s). The WtR recovery was significantly slower in old muscles (τ_r_ = 41.4 s) than in young muscles (τ_r_ = 15.4 s).

**TABLE 3:** Time constants of interstitial PO_2_ transients in young and old rat muscles.

Curve fitting parameters (s)	Young (*N* = 117)	Old rats (*N* = 139)
Lag period s	4	4
τ_w_ s; work, #11–60	9.0 ± 3.7	15.9 ± 3.5*
τ_r_ s; rest, #101–250	15.4 ± 3.9	41.4 ± 8.3*

*Note*: Data are presented as mean ± SD. *N* represents the number of measurements in muscle sites.

Abbreviations: τ_r_, time constant for work‐to‐rest transition; τ_w_, time constant for rest‐to‐work transition.

* *p* < 0.001 between mean values.

During the RtW transition in young muscles, reaching the nadir was followed by a slow positive trend of PO_2_ of about 0.1 mmHg/s (Figure 2, dashed line). Therefore, the lowest point was taken as characteristic *P*_w_, which also agreed well with the exponential fitting. Interestingly, the RtW transition was protracted in old muscles when reaching the nadir, and no subsequent positive trend was detected. The fastest RtW transient was observed in young, while it was almost 50% slower in old (Table 3). Overall, the WtR transients were much slower than the RtW transients, especially in old (Table 3).

### 3.4 Intra‐contractile PO_2_ spike in young muscles

**FIGURE 3:**
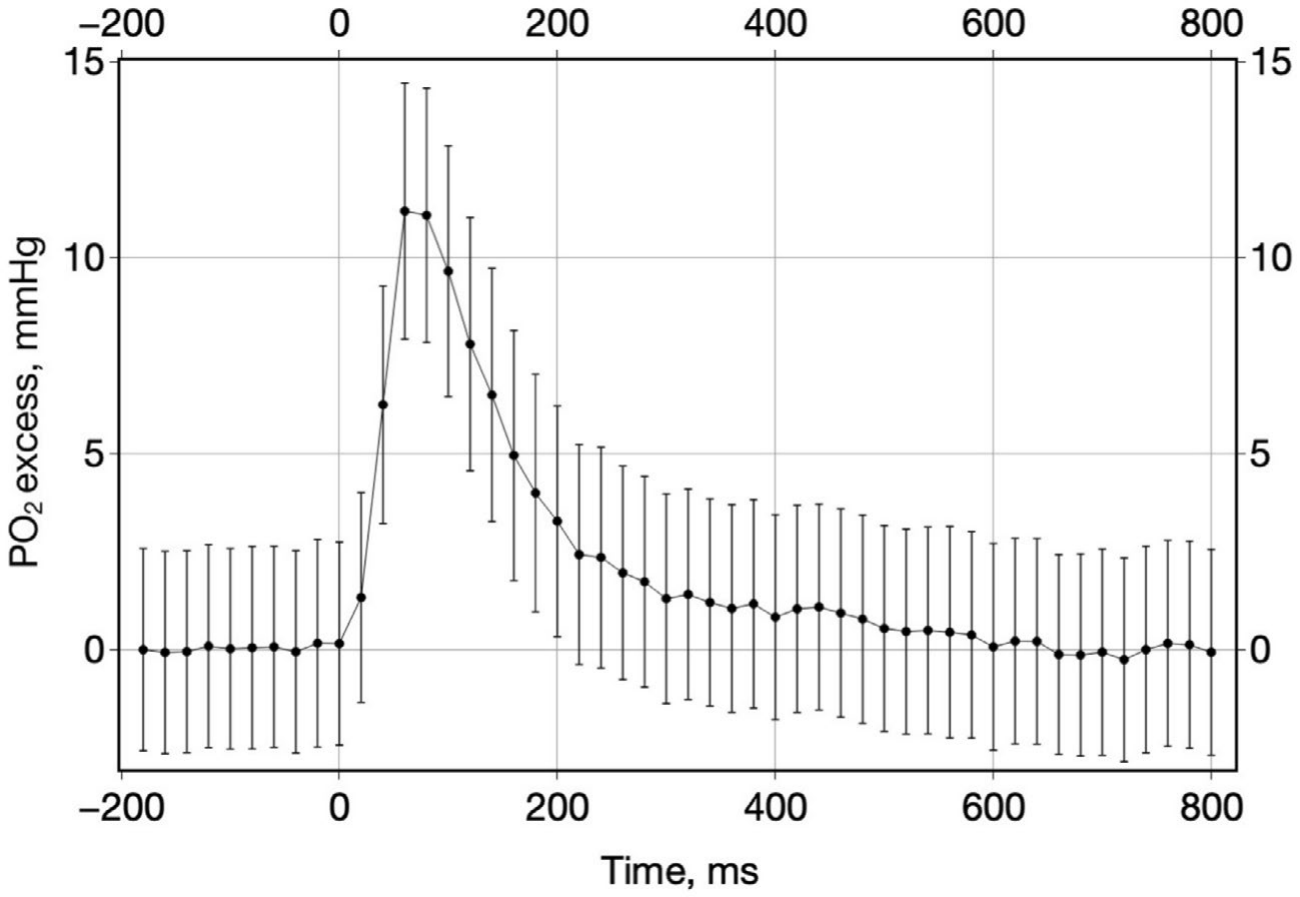
Contraction‐induced PO_2_ spike in young rat muscle. High temporal resolution measurement of the PO_2_ spike during muscle contraction in young rat spinotrapezius muscle. Data were compiled from 40 consecutive contractions using the stroboscopic method, averaging 117 measurements per every time point. The spike starts at point 0 and reaches an amplitude of 11.1 mmHg within 80 ms and then decays mono‐exponentially with a time constant of 99.5 ms. Data are presented as mean ± CI 95%.

The stroboscopic measurement technique revealed a distinct PO_2_ spike embedded within the muscle contractions of young rats (Figure 3). This spike, with an amplitude of 11.1 mmHg and a duration exceeding 200 ms, was absent in old muscles. The PO_2_ spike in young rats was superimposed on a positive linear trend over time: (*P*_i_ = 37.7 + 0.096 * *t*).

In the previous report on the PO_2_ spike (Golub et al., 2021), the spike was not corrected for a linear trend. In the present data set (Young), a linear trend was subtracted from the spike segment of the data before the rate of PO_2_ changes was estimated. After correcting, the PO_2_ spike showed a mono‐exponential decay with a time constant of 99.5 ms (Figure 3).

## 4 DISCUSSION

### 4.1 Age‐related differences in skeletal muscle oxygenation

These studies were carried out on two animal groups of contrasting ages, 3 and 23 months, which are reported to be similar to humans aged under 18 and over 60 years (Sengupta, 2013). Another similarity is that laboratory animals are less active over their lifespans than their wild counterparts, which may better reflect the pathologies emergent in sedentary human populations as they age. Our findings demonstrate significant age‐related differences in skeletal muscle oxygenation dynamics and reveal a loss of an intra‐contractile PO_2_ “spike” that may play a fundamental role in aerobic muscle function.

The lower baseline and nadir PO_2_ values observed in old rat muscles (Table 3) align with previous studies reporting decreased microvascular PO_2_ in aged skeletal muscle (Behnke et al., 2005; Russell et al., 2003). This reduction in muscle oxygenation can be attributed to two primary factors: (1) Decreased arterial blood PO_2_ in aged rats. This systemic change likely contributes substantially to the observed decrease in muscle oxygenation. (2) Alterations in muscle microvasculature: Previous studies have reported age‐related decreases in capillary density and blood flow (Muller‐Delp, 2016; Payne & Bearden, 2006), which may further compromise O_2_ delivery to muscle tissue.

Clinical data from various age groups in humans support the hypothesis of decreased arterial PO_2_ with age (Blom et al., 1988; Delclaux et al., 1994; García‐Río et al., 2007). Initially, these findings did not appear translatable to rats as blood gas data from sedentary and trained Fischer 344 rats aged 6 and 24 months showed high blood oxygenation levels of 92–94 mmHg, independent of age (McCullough et al., 2011). However, studies conducted on Sprague–Dawley male rats over their lifespan revealed a significant difference in arterial blood PO_2_ between 3‐month‐old and 24‐month‐old animals. The arterial PO_2_ levels for 3‐month rats were 91.9 ± 8.0 (11 rats) mmHg and 81.3 ± 8.0 (12 rats) mmHg for 24‐month‐old rats, with a significant difference of 10.6 mmHg (*p* < 0.01; (Nugent et al., 2025)), which agrees with the age differential baseline interstitial PO_2_ of 6.5 mmHg found in this study (Table 2).

The adaptive aging hypothesis proposes maintaining a balance between O_2_ supply and consumption, so interstitial PO_2_ changes with age in proportion to the decrease in arterial oxygenation. Basal PO_2_ in the muscles of young and old rats (Table 2) is 73%–74% of the PO_2_ in their arterial blood, which supports the hypothesis. However, under load, a disproportion occurs; PO_2_ in the muscles of young rats is 46% of the arterial, but in old rats only 35%. Since mitochondrial respiration is known to decrease with age, the relatively low muscle PO_2_ can only be associated with a limitation of O_2_ delivery by microvessels. Therefore, if age‐related adaptation occurs, then it is primarily attuned to the resting state. During muscular work, limitations in O_2_ delivery lead to a disproportionate decrease in tissue oxygenation.

The data's interpretation is complicated by the rate of cellular respiration's dependence on interstitial PO_2_, leading to a decrease in O_2_ consumption similar to the functional decline in mitochondria (Pittman, 2011). An accurate analysis of the interdependence of O_2_ delivery and consumption systems requires a mathematical model based on experimental data.

### 4.2 Experimental transients

The onset and cessation of muscle contraction cause adaptation of its O_2_ consumption, which is reflected in the PO_2_ transients in the interstitium and microvessels. The RtW transient is much faster than the WtR transient, and this asymmetry is of particular interest.

Experimental records of PO_2_ in young and old muscles contain segments with RtW and WtR transients, which are described by mono‐exponential curves of asymptotic decline and gain. Both groups have a lag period of 4 s, a phenomenon well known as a “time delay” (Hirai et al., 2018) or a “lag period” (Wilson, 2015). In our experiments, the RtW transitions occurred 2–3 times faster than the PO_2_ recovery during the WtR process.

The rapid fall in PO_2_ at RtW means a sharp increase (just after the lag period) in the respiratory rate of myocyte mitochondria, which decreases as a new balance of O_2_ supply and consumption is reached. At the same time, the rapid activation of cellular respiration does not exclude the existence of a slow transient in VO_2_ measured in the whole organ using the steady‐state Fick principle (Behnke et al., 2003). The switching of the WtR transient is also instantaneous, within 1 s, and the ascending transients are also mono‐exponential (Table 3, Figure 2).

Considering the submicron distances between the elements of oxidative phosphorylation in the mitochondrial membrane, one can estimate diffusion and transport delays at the millisecond range; it is difficult to accept the idea that the activation of oxidative phosphorylation takes tens of seconds to minutes (Korzeniewski, 2004; Wilson, 2015). Our data are consistent with the classic conclusion that “The initiation of increased respiration of mitochondria following ADP addition requires less than 1 second and is rapid enough to explain measured responses of respiration to physiological activity.” (Chance & Williams, 1955).

In addition, we can note simple relationships that cross‐link the main parameters of RtW and WtR transients: *P*_r_* τ_w_ = *P*_w_* τ_r_; (Figure 1, Tables 2, 3). For young muscles, this product is equal to 650 and 654; for old muscles, it is 1038 and 1172. Calculation from the literature data on interstitial PO_2_ transients obtained in hypoxic muscles (Hirai et al., 2019) yielded results of 274 and 290. The ratios are close enough to support the hypothesis that the speed of transients of interstitial PO_2_ is governed by simple physical principles, not metabolic ones.

### 4.3 Intracontractile PO_2_ spike: A phenomenon in young but not in old muscle

The phenomenon of a fast PO_2_ spike during the contraction of the rat spinotrapezius muscle of young rats was first reported in 2021 (Golub et al., 2021). The onset of this PO_2_ spike coincided with the onset of muscle contraction, with an 11 mmHg peak and a duration of over 200 ms (Figures 2 and 3). It has been attributed to mechanical effects on blood flow caused by the intramuscular pressure pulse produced by contraction. In concert, a constant positive rise in interstitial PO_2_ was observed throughout muscular work. A similar effect of rising oxygenation was previously reported in several muscles and was interpreted as “PO_2_ undershoot” (Poole & Ferreira, 2007).

In the present work, it was found that old muscles lack both PO_2_ spikes and the positive PO_2_ trend. We hypothesize that the PO_2_ spike and the positive PO_2_ trend are related effects and speculate that young muscles benefit from a small, compounding O_2_ inflow during each contraction from the PO_2_ spikes. A positive shift in the balance point between O_2_ delivery and consumption due to the proposed mechanism may have functional significance during prolonged muscular work.

Regarding the spike's mechanism, we suggest it is a consequence of a muscle contraction‐induced burst of blood flow through capillaries. The absence of the PO_2_ spike and positive PO_2_ trend in old muscles indicates a possible relationship between the pumping mechanism and the arterial elasticity that is degraded in older animals. In this case, there is a return to the historical muscle pump hypothesis but with more O_2_ for the muscle itself than for increased total blood flow.

Our high‐temporal resolution measurements of muscle PO_2_ dynamics reveal several novel findings that enhance our understanding of muscle aging. First, we discovered a contraction‐induced PO_2_ spike in young muscle, which is absent in older muscle. This suggests that there is an age‐sensitive mechanism for increasing oxygen delivery during physical activity.

Second, we observed distinct patterns of PO_2_ transients between the age groups. Notably, the differences in time constants and the presence or absence of positive trends during sustained work indicate fundamental changes in how aging muscle manages its oxygen supply and demand.

While these findings can support multiple interpretations of the underlying mechanisms, they provide essential experimental evidence for testing theoretical models of muscle oxygen dynamics. The precise quantification of rest‐work and work‐rest transitions, coupled with the discovery of rapid intra‐contractile oxygen dynamics, establishes new constraints for understanding how aging affects the integration of vascular and metabolic functions in skeletal muscle.

### 4.4 Estimated diffusion relaxation of PO_2_ spike

PO_2_ measurements in the stroboscopic mode were made every second and superimposed on the positive trend of PO_2_, which distorted the shape of the PO_2_ spike. The PO_2_ trend line was approximated by the function *P*_i_ = 37.7 + 0.096 *t* (time, s) and subtracted from the PO_2_ spike curve (Figure 2). The corrected spike has an amplitude of 11.1 mmHg and a mono‐exponential decay with a time constant of 99.5 ms (Figure 3). This mono‐exponential fall can be represented as diffusional relaxation of a brief injection of oxygenated blood into the capillaries, caused by muscle contraction. The exponential lifetime can be used to approximate the diffusion coefficient of O_2_ using Einstein's equation:

$$\left\langle x^{2}\right\rangle =2\mathrm{Dt}$$

where *x* is the mean square displacement, *D* is the diffusion coefficient, and t is the characteristic time. If *x* is half the distance between the capillaries (*x* = 1.7•10^−3^ cm) (Gray, 1973), and the time constant (*t* = 0.0995 s) is the diffusion time of O_2_ from the capillaries, then the estimated O_2_ diffusion coefficient in muscle *D* = 1.45•10^−5^ cm^2^/s, which is consistent with many reported estimates in hamster muscles (Bentley et al., 1993; Ellsworth & Pittman, 1984). Good agreement with the diffusion coefficient in skeletal muscle supports the hypothetical mechanism of O_2_ spike formation during muscle contraction. A positive shift in the balance point between O_2_ delivery and consumption due to the proposed mechanism may have functional significance during prolonged muscular work.

The absence of the PO_2_ spike and positive PO_2_ trend in old muscles indicates a possible relationship between the pumping mechanism and the arterial elasticity that is degraded in older animals. In this case, there is a return to the historical muscle pump hypothesis; only in this case, more for O_2_ in the muscle itself than for increased total blood flow.

### 4.5 Implications for muscle aging theories

Our findings have implications for both the vascular and mitochondrial theories of muscle aging: (1) Vascular theory: The observed decreases in baseline interstitial PO_2_ and slower O_2_ kinetics in old muscles support the concept of impaired O_2_ delivery due to age‐related vascular changes. Research has indicated that there is a reduction in the number of feeding arterioles and capillary density in the muscles of older rats, which contributes to a decrease in interstitial oxygen partial pressure (Landers‐Ramos & Prior, 2018; Muller‐Delp, 2016). (2) Mitochondrial theory: While our study did not directly measure mitochondrial function, the absence of the PO_2_ spike and slower O_2_ kinetics in old muscles could reflect compensatory reductions in mitochondrial capacity to increase O_2_. Additionally, the decrease in interstitial oxygen tension in aged muscles is likely linked to an increased oxygen demand. This demand is associated with oxygen leaks resulting from elevated formation of reactive oxygen species and mitochondrial uncoupling, which occurs due to increased proton leakage and decreased efficiency of oxidative phosphorylation (Amara et al., 2007; Jackson & McArdle, 2011). The slower O_2_ kinetics observed in old muscles during both rest‐to‐work (RtW) and work‐to‐rest (WtR) transitions (Table 3) suggest impaired O_2_ delivery and/or utilization with age. These findings support the vascular theory of muscle aging (Ungvari et al., 2010) and indicate a reduced capacity for rapid adjustments to changes in metabolic demand.

The complex interactions between oxygen delivery and utilization cannot be fully understood through isolated measurements of blood flow, mitochondrial function, or muscle performance alone. Our approach of measuring interstitial PO_2_ dynamics across multiple time scales reveals emergent properties of the integrated system. The age‐related loss of the PO_2_ spike phenomenon, combined with altered rest–work transition kinetics, suggests that aging affects not just the steady‐state balance of oxygen supply and demand, but also the dynamic coordination between vascular and metabolic responses. These findings bridge the gap between structural studies of muscle aging and functional measurements of muscle performance, providing new mechanistic insights into how aging compromises muscle adaptability.

## 5 CONCLUSIONS

This study offers insights into age‐related changes in muscle function through depressed skeletal muscle oxygenation dynamics and loss of a novel, intra‐contractile oxygen pump. Muscle oxygenation and utilization decline with age due to decreases in arterial PO_2_ and microvasculature, and biomechanics of muscle showing preferential adaptation to the resting state. Also, exercise on‐kinetics are slower and faster to exhaust local PO_2_. Rather than a biochemical mechanism, a biophysical mechanism is proposed involving the loss of a recently described “spike” in PO_2_, which could be fundamental to maintaining aerobic muscle function. Finally, it is clear the current descriptive analysis of experimental data on skeletal muscle PO_2_ response to exercise is insufficient to fully understand the regulatory relationships and mechanisms. A mathematical model must be applied to comprehend the relationship between O_2_ delivery and consumption for a more thorough interpretation of PO_2_ transients and steady‐state levels.

## AUTHOR CONTRIBUTIONS

A.S.G., R.N.P., and B.K.S. conceived and designed research; A.S.G. and B.K.S. performed the experiment. A.S.G., W.H.N., and B.K.S. analyzed data; A.S.G., W.H.N., R.N.P., and B.K.S. interpreted results of experiments; A.S.G. prepared figures; A.S.G. and W.H.N. drafted the manuscript; A.S.G., W.H.N., R.N.P., and B.K.S. edited and revised the manuscript, approved the final version of the manuscript.

## FUNDING INFORMATION

This study was funded and performed by Song Biotechnologies, LLC.

## CONFLICT OF INTEREST STATEMENT

Aleksander Golub, William Nugent, and Bjorn Song are employees of Song Biotechnologies, LLC, which was responsible for all study costs. Roland Pittman received no compensation for his contributions and has no conflicts of interest to declare.

## Data Availability

For original data, please contact bjorn@songbiotechnologies.com.
